# The Reconstitution of the Macrophage Niche Reveals Dynamic Transcriptional and Renal Macrophage–Epithelial Communication Networks

**DOI:** 10.3390/cells15121102

**Published:** 2026-06-18

**Authors:** Mohammad Islamuddin, Lixuan Ji, Yilin Chen, Kejing Song, Calder R. Ellsworth, Jack Rappaport, Chenxiao Wang, Shumei Liu, Jay Kolls, Xiaojiang Xu, Xuebin Qin

**Affiliations:** 1 Tulane National Biomedical Research Center, Covington, LA 70433, USA; mislamuddin@tulane.edu (M.I.); ychen85@tulane.edu (Y.C.); cellsworth@tulane.edu (C.R.E.); cwang27@tulane.edu (C.W.); sliu32@tulane.edu (S.L.); 2 Department of Microbiology and Immunology, Tulane University School of Medicine, New Orleans, LA 70112, USA; jackrapp@me.com; 3 Department of Pathology and Laboratory Medicine, Tulane University School of Medicine, Tulane University, New Orleans, LA 70112, USA; lji1@tulane.edu (L.J.); xxu17@tulane.edu (X.X.); 4 Departments of Medicine and Pediatrics, Center for Translational Research in Infection and Inflammation, Tulane University School of Medicine, New Orleans, LA 70112, USA; ksong1@tulane.edu (K.S.); jkolls1@tulane.edu (J.K.); 5 Department of Pulmonary Critical Care and Environmental Medicine, Tulane University School of Medicine, New Orleans, LA 70112, USA

**Keywords:** renal macrophages, niche regeneration, proximal tubule epithelial cells, epithelial–immune crosstalk, cell–cell communication networks, osteopontin

## Abstract

**Highlights:**

**What are the main findings?**

- RM regeneration is controlled by epithelial CX3CL1 signaling, macrophage metabolic reprogramming, and a conserved transcriptional network.
- Spp1 (osteopontin)–mediated macrophage–epithelial communication drives niche restoration and tubular repair after depletion.

**What are the implications of the main findings?**

- Defines an epithelial–immune circuit governing macrophage regeneration and kidney repair.
- Identifies CX3CL1, Spp1, and metabolic pathways as potential therapeutic targets for renal regeneration.

**Abstract:**

Renal-resident macrophages (RMs) are essential regulators of kidney homeostasis and repair, yet the mechanisms governing RM niche regeneration after acute depletion remain poorly defined. To overcome these limitations, we have developed an inducible human CD59- intermedilysin (hCD59-ILY) ablation system, enabling rapid, specific, and reversible depletion of targeted macrophage populations, and subsequent replenishment of RMs, followed by longitudinal scRNA-seq analysis of kidneys at baseline and days 1, 3, and 7 post-ablation. RM ablation triggered a rapid and sustained upregulation of *Cx3cl1*, predominantly in proximal tubular epithelial cells (PTC1/PTC2), establishing a persistent chemotactic niche signal that coincided with macrophage repopulation. Regenerating RMs transitioned from inflammatory/stress-associated states toward metabolically active and proliferative phenotypes enriched in glycolysis, oxidative phosphorylation, MYC, and cell-cycle programs, with attenuation of canonical inflammatory pathways. Cell–cell communication analysis revealed an early burst of intercellular signaling at day 1, followed by progressive normalization, with fibronectin *(Fn1*), osteopontin (*Spp1*), chemokine (*Ccl*), and amyloid precursor protein (*App*) axes emerging as key mediators of niche restoration. Transcriptional network analysis identified a conserved regulatory module (*Tfe3*, *Mitf*, *Hif1a*, *Myc*, *Gabpa*, *Rcor1*) coordinating macrophage differentiation and regenerative programming, linking metabolic adaptation to lineage reconstitution. Sub-clustering revealed five dynamically shifting RM subsets with distinct inflammatory, remodeling, proliferative, and surveillance states, reflecting a hierarchical regeneration process. Functional validation using clodronate-mediated depletion in Secreted Phosphoprotein 1 *(Spp1)* (Opn)-deficient mice demonstrated impaired macrophage repopulation, establishing osteopontin as a critical regulator of RM regeneration. Together, these data define a coordinated epithelial–immune circuit in which Cx3cl1-driven chemotaxis, *Spp1*-dependent signaling, and a core transcriptional network orchestrate macrophage niche reconstitution and kidney repair following acute immune cell ablation.

## 1. Introduction

Renal macrophages (RMs) play indispensable roles in maintaining kidney homeostasis, orchestrating immune surveillance, and mediating responses to injury and repair [1,2]. These cells exist as a heterogeneous population derived from embryonic yolk sac progenitors, fetal liver monocytes, and bone marrow-derived monocytes, contributing to distinct resident and infiltrating macrophage pools [3,4,5,6]. Their maintenance and phenotype are tightly regulated by local cues from tubular epithelial cells, endothelial cells, and extracellular matrix (ECM) components [7,8]. Following kidney injury, macrophages act as central effectors of inflammation, resolution, and regeneration, dynamically shifting from pro-inflammatory to reparative states [9,10,11]. This phenotypic versatility enables RMs to coordinate immune cell recruitment, matrix remodeling, and epithelial recovery [12]. Recent advances in single-cell RNA sequencing (scRNA-seq) have revolutionized our understanding of RM heterogeneity, revealing distinct subsets such as Ccr2^+^Ly6C^+^ inflammatory macrophages, Fn1^+^ remodeling macrophages, and Pglyrp1^+^Ace^+^ immunomodulatory cells [12,13,14,15,16,17,18]. These subsets engage in temporally structured communication with proximal tubular epithelial (PTE) cells and other immune populations through defined ligand–receptor pairs, most notably Fn1-Cd44, Fn1-Sdc4 and (Secreted Phosphoprotein 1) Spp1-Cd44, to coordinate ECM remodeling, immune regulation, and epithelial regeneration [17,18,19,20]. At the transcriptional level, repair-associated regulators such as *Klf4*, *Irf8*, and *E2f* families integrate microenvironmental signals (hypoxia, cytokines, and metabolic stress) to modulate macrophage polarization, proliferation, and reparative signaling [21,22,23,24]. Macrophage-derived growth factors, including *Csf-1* and *Spp1* (osteopontin), further promote tubular cell proliferation and differentiation, underscoring macrophage epithelial reciprocity in renal repair [25,26,27]. Despite these insights, the temporal and mechanistic hierarchy linking macrophage depletion, niche repopulation, transcriptional activation, and intercellular communication during kidney injury remains undefined.

Addressing this knowledge gap requires a system that can acutely and specifically perturb the macrophage compartment while preserving the renal microenvironment for unbiased molecular interrogation. Historically, experimental macrophage depletion strategies, including clodronate liposomes, irradiation, and diphtheria toxin receptor (DTR) models, have provided mechanistic insights into macrophage biology but are constrained by incomplete ablation, off-target toxicity, and compensatory hematopoietic activation [28,29,30]. To overcome these limitations, we have developed an inducible human CD59-intermedilysin (hCD59-ILY) ablation system, enabling rapid, specific, and reversible depletion of targeted macrophage populations [5,31,32]. The ILY toxin from Streptococcus intermedius binds exclusively to hCD59, forming pores that lyse expressing cells within seconds, allowing temporally controlled depletion [32,33]. This unique system facilitates dynamic tracking of macrophage regeneration, offering a clean platform to probe the transcriptional and cellular events underpinning RM niche restoration. Here, we employed our hCD59-ILY ablation platform in combination with high-throughput scRNA-seq and cell–cell communication mapping to characterize the temporal kinetics, transcriptional architecture, and signaling hierarchy underlying RM depletion and repopulation. We demonstrated temporally distinct RM subsets and signaling pathways, including *Spp1-Cd44* and *Fn1*-integrin axes that orchestrated immune–epithelial crosstalk during niche restoration and linked 18 upregulated transcription factors to interleukin-1 responses, myeloid differentiation, and tissue remodeling for a transcriptionally coordinating RM regeneration.

## 2. Materials and Methods

### 2.1. Animal Model Strains and Their Maintenance

*Cx3cr1CreER^+/+^* mice (JAX 021160) were obtained from The Jackson Laboratory (Bar Harbor, ME, USA) and housed at the Tulane University School of Medicine. The *ihCD59^+/+^* mice, previously generated and backcrossed onto a C57BL/6 genetic background for at least seven generations [32], were used for breeding. Homozygous *Cx3cr1CreER^+/+^* mice are deficient in CX3CR1 expression (Cx3cr1 knockout), whereas heterozygous *Cx3cr1CreER^+/−^* mice retain functional CX3CR1 expression. To generate experimental genotypes, *Cx3cr1CreER^+/+^* mice were crossed with *ihCD59^+/+^* mice to produce the following offspring: *Cx3cr1CreER^+/−^/ihCD59^+/−^*. All animal experiments were conducted in accordance with the ethical guidelines of the Institutional Animal Care and Use Committee (IACUC) at Tulane University (Protocol number 1482). Mice were housed in a specific pathogen-free (SPF) facility at the Tulane University School of Medicine, maintained under a 12 h light/dark cycle with controlled environmental conditions. Detailed experimental protocols, including intermedilysin purification, tamoxifen treatment, RM depletion and regeneration, sample collection schedules, flow cytometry staining and acquisition. RNA isolation and qRT-PCR, scRNA-Seq by 10× genomics and data analysis, and statistical analysis are provided in the Supplementary Materials.

### 2.2. Intermedilysin Purification

Recombinant His-tagged intermedilysin (ILY) was purified using the HisBind Purification Kit (EMD 70239; Novagen, Merck Millipore, Darmstadt, Germany) following the manufacturer’s instructions, as previously described [31,32]. Briefly, bacterial cultures expressing His-tagged ILY were lysed, and the protein was captured using a nickel-affinity chromatography column. After washing to remove non-specifically bound proteins, the His-tagged ILY was eluted under optimized conditions. The concentration and purity of the purified ILY were assessed by SDS-PAGE, ensuring adequate purity for downstream applications.

### 2.3. Tamoxifen Treatment, RM Depletion, and Regeneration

Tamoxifen (Sigma-Aldrich; St. Louis, MO, USA) was dissolved in corn oil at a concentration of 20 mg/mL. To induce Cre-mediated hCD59 expression in *Cx3cr1CreER^+/−^/ihCD59^+/−^* mice, 10–12-week-old mice were administered tamoxifen at a dose of 100 μg/g body weight via intraperitoneal (i.p.) injection for 3 consecutive days. After a 15-day waiting period post-tamoxifen treatment, *Cx3cr1CreER^+/−^/ihCD59^+/−^* mice and *Cx3cr1CreER^+/−^/ihCD59*^−*/*−^ littermate controls were injected with a single dose of intermedilysin (ILY) at 120 ng/g body weight. RM ablation and subsequent regeneration were monitored and confirmed by flow cytometry at day ‘0’ (D0) day ‘1’ (D1), day ‘3’ (D3) and day ‘7’ (D7) post-ILY administration [5,6].

Of note, previously we have demonstrated that ILY does not have any effect on the macrophage depletion population in *ihCD59*^−/−^ littermate control [5]. Moreover, toxicity experiments revealed that acute and chronic in vivo treatment with a 10- to 20-fold ILY dose that was higher than the effective dose for cell ablation did not cause any detectable toxicity in Cre-negative ihCD59+ mice, further confirming that ILY has a large pharmaceutical window without any off-target effects [32]. Because of the specific effect of ILY on hCD59 labeled cells in the mice with a large pharmaceutical window, together with scRNA-seq cost limitation, we did not include the Tamoxifen and ILY treated-*ihCD59*^−/−^ littermate control.

### 2.4. Preparation of Single Cells from Mouse Tissue

Mice were euthanized using carbon dioxide (CO_2_) asphyxiation followed by perfusion with phosphate-buffered saline (PBS) to remove blood from tissues. Kidneys were harvested post-perfusion and processed for single-cell suspension preparation. Tissue dissociation was initiated by mechanical disruption of the kidney, followed by enzymatic digestion. The dissected kidney was incubated in 10 mL of Hank’s Balanced Salt Solution (HBSS), containing 0.75 mg/mL collagenase IV (Worthington Biochemical; Lakewood, NJ, USA) and 20 µg/mL DNase I (Sigma-Aldrich; St. Louis, MO, USA), for 40 min at 37 °C. After digestion, the cell suspension was passed through a 40 µm cell strainer to remove debris and larger tissue fragments, and the resulting single-cell suspension was maintained on ice. The cell suspension was then washed twice with PBS by centrifugation at 500× *g* for 10 min. To enrich hematopoietic cells, the cells were subjected to density gradient centrifugation using Percoll. The pellet obtained from the previous wash was resuspended in 10 mL of 30% Percoll solution and carefully layered over 3 mL of 70% Percoll solution. The gradient was centrifuged at 500× *g* for 30 min without the application of a brake. Following centrifugation, the interphase layer between the 30% and 70% Percoll solutions, containing enriched single cells, was carefully collected. The collected cells were washed twice with 10 mL of PBS (500× *g*, 10 min). The final cell pellet was resuspended in RPMI-1640 medium supplemented with 10% fetal bovine serum (FBS) for downstream single-cell RNA sequencing (scRNA-seq) analysis. Cell number and viability were assessed using an automated cell counter, yielding a viability rate of approximately 84%, suitable for scRNA-seq. Simultaneously, a portion of the cells post-density gradient centrifugation was prepared for flow cytometry. The cells were washed twice with 10 mL of PBS (500× *g*, 10 min) and then resuspended in 100 µL of flow cytometry staining buffer (PBS with 2% FBS). The suspension was further processed for antibody staining following standard protocols for flow cytometric analysis [5].

### 2.5. Clodronate Liposome-Mediated Depletion and Repopulation Analysis of RMs in Wild-Type and Osteopontin Knockout Mice

Ten- to twelve-week-old C57BL/6 wild-type (B6 WT) and osteopontin knockout (*Opn−/−*) mice were used in all experiments. To deplete RMs, mice received an intraperitoneal (i.p.) injection of 200 µL clodronate liposomes (LIPOSOMA, Amsterdam, The Netherlands). Control animals received an equal volume of PBS. Mice were sacrificed at 2, 7 and 9 days post-injection (DPI) to assess depletion and repopulation, respectively. Kidneys were perfused with PBS, minced, and digested with collagenase D and DNase I for 30 min at 37 °C. RMs were identified as CD45^+^ CD11b^+^ F4/80hi by flow cytometry.

### 2.6. Flow Cytometry Staining, Acquisition

The single-cell suspensions prepared from mouse kidney tissue were processed for flow cytometric analysis. To block non-specific binding to Fc receptors, cells were incubated with anti-CD16/32 antibody (FcγRIII/II, Clone 93, Cat# 48-0161-80, eBioscience, San Diego, CA, USA) at a dilution of 1:200 for 15 min at room temperature. Aqua live/dead dye (Cat# L34957A; Invitrogen, Carlsbad, CA, USA) was used to distinguish live from dead cells according to the manufacturer’s instructions. For phenotypic analysis of RM ablation, cells were stained with the following pre-conjugated antibodies at a 1:100 dilution: CD45-e450 (Clone 30-F11, Cat# 48-0451-82; eBioscience, San Diego, CA, USA), CD11b-PE-Cy7 (Clone M1/70, Cat# 25-0112-82; Invitrogen, Carlsbad, CA, USA), hCD59-PE (Clone OV9A2, Cat# 12-0596-42; Invitrogen, Carlsbad, CA, USA), and F4/80-BV605 (Clone BM8, Cat# 123133; BioLegend, San Diego, CA, USA). The antibody cocktails were added to the cell suspensions, and samples were incubated for 30 min at 4 °C in the dark to protect fluorophores from photobleaching. Following staining, the cells were washed twice with flow cytometry buffer (PBS containing 2% fetal bovine serum). Cells were then fixed in 1% paraformaldehyde (PFA) for 30 min on ice, followed by two additional washes with flow cytometry buffer. The stained and fixed cells were acquired using a BD LSR Fortessa flow cytometer, and data were analyzed using FACSDiva software, version 6.1.3. (BD: https://www.bdbiosciences.com/en-ca/products/instruments/softwareinformatics/instrument-software/bd-facsdiva-software-v-6-1-3.643629, accessed on 30 September 2025).

### 2.7. RNA Isolation and qRT-PCR

Total RNA was isolated from mouse kidney tissue using the RNeasy Plus Mini Kit (Cat#74134; Qiagen, Hilden, Germany) following the manufacturer’s protocol. RNA integrity and quantity were assessed using spectrophotometric analysis. For reverse transcription, 1 µg of RNA was used as input for cDNA synthesis, performed with the High-Capacity cDNA Reverse Transcription Kit (Cat# 4368814; Applied Biosystems, Foster City, CA, USA) [11]. The reaction was carried out according to the manufacturer’s instructions. Quantitative PCR (qPCR) was conducted on the synthesized cDNA using Perfecta SYBR Green FastMix Low ROX (Cat# 95074-250; QuantaBio, Beverly, MA, USA) as the detection chemistry. Amplification was performed using the QuantStudio 3 Real-Time PCR System (Thermo Fisher Scientific, Waltham, MA, USA). The cycling conditions included an initial denaturation at 95 °C for 30 s, followed by 40 cycles of denaturation at 95 °C for 5 s and annealing/extension at 60 °C for 5 s. Target gene expression levels were normalized to the housekeeping gene beta-actin. The relative expression levels of target genes were calculated using the 2^−ΔΔCT^ method. The following qPCR primers were used, synthesized by Integrated DNA Technologies (IDT, Coralville, IA, USA): *Cx3cl1* (forward: 5′-GGACAAGCCACATAGGAAAGA, reverse: 5′-CACATGCACAAGTCCCTACA). Gene expression data were analyzed and plotted to determine relative fold changes in mRNA levels.

### 2.8. scRNA Sequencing by 10× Genomics

scRNA-seq was performed using the 10× Genomics Chromium platform (10× Genomics, Pleasanton, CA, USA) to analyze transcriptomic profiles of kidney cells. A target of 5000 viable cells per sample was set based on prior optimization using 10× Genomics Single Cell 3′ RNAseq technology (10× Genomics). Live cells were quantified and sorted before processing, ensuring the cell viability exceeded 80% for downstream analysis. Following cell capture, full-length barcoded cDNA libraries were generated from each sample according to the manufacturer’s protocol. Briefly, individual cells were partitioned into droplets containing reagents for reverse transcription, generating unique cDNA barcodes for each cell. The cDNAs were then amplified via polymerase chain reaction (PCR) to obtain sufficient material for library preparation. The cDNA libraries were pooled and diluted to a final concentration of 1.8 pM for sequencing. The pooled libraries were sequenced using an Illumina NextSeq 2000 platform (Illumina, San Diego, CA, USA, with paired-end single index reads. Sequencing output was analyzed to ensure adequate coverage and depth for each sample. Raw sequencing data were processed using Cell Ranger version 7.1.0 (10× Genomics) to perform alignment to the mouse reference genome, read counting, and the generation of cell-specific gene expression matrices. To identify differentially expressed genes across cell clusters, Loupe Cell Browser 8.12 (10× Genomics) was used.

### 2.9. scRNA Data Cell Identification and Annotation of Cell Clusters

We performed a standard sequence of filtering, where highly variable gene selection, dimensionality reduction, and clustering were performed using the scRNA-seq analysis R (version 4.3.0)-based package Seurat (version 5.0.0) for quality control and downstream analysis. After alignment and initial preprocessing, we began our workflow with a total of 32,285 genes across 31,100 cells from single-cell RNA sequencing (scRNA-seq) on the 10× Genomics Chromium Platform using kidneys from *Cx3cr1CreER^+/−^/ihCD59^+/−^* mice treated with ILY. For the next QC process, we filtered out cells with gene counts (nFeature) of less than 50 and greater than 8000 and with a UMI (nCount) of greater than 40,000 to remove low quality cells and possible multiple captures. We also eliminated low-quality cells with greater than 50% mitochondrial gene expression. A total of 21,181 genes across 27,396 single cells were included in the downstream analysis after applying the quality control criteria, which include 19,564 genes across 4782 cells in Normal kidney group, 19,479 genes across 5004 cells in D0 group, 19,161 genes across 5216 cells in D1 group, 19,358 genes across 5830 cells in D3 group, and 19,341 genes across 6564 cells in D7 group. Normalization was performed using the Seurat package to reduce biases introduced by technical variation, sequencing depth, and capture efficiency. A correlation analysis and principal component analysis were performed and uniform manifold approximation and projection (UMAP) was used to classify cells into different cell clusters at a proper resolution. Batch effects were corrected using “rPCA” from Seurat. Highly variable genes were identified by iterative selection based on the dispersion versus average expression of these genes. Principal component analysis was used for dimension reduction, and the top 30 principal components were selected by a permutation-based test implemented in Seurat and passed to UMAP to visualize cell clusters. Cell cluster annotation was conducted based on the known biomarkers of different cells in the kidney as published in previous studies [9,34,35,36]. DEGs among different clusters were identified to validate the reasonableness of cell cluster annotation.

### 2.10. Identification of DEG and Hallmark Pathways Using Gene Set Enrichment Analysis

Differentially expressed genes (DEGs) were identified using the FindMarkers function in Seurat (test.use = Mast). Adjusted *p*-value < 0.01, min.pct = 0.01, and logfc.threshold = 0.01 were used as thresholds for significant differential expression. Gene Set Enrichment Analysis (GSEA) was then performed via the GSEA function in the package clusterProfiler (4.4.4) to clarify the of RM recruitment and dynamic biological change in different experiment time points based on DEG. Human MSigDB Collections (https://www.gsea-msigdb.org/gsea/msigdb/mouse/collection_details.jsp, accessed on 3 June 2026) is utilized as a database. To identify genes with consistent transcriptional changes across experimental conditions, differential expression results from each cell population (Macro/PTC1/PTC2/Cx3cr1^+^ cells) were processed as follows. First, low-confidence or predicted genes were removed by excluding gene symbols beginning with Gm-, mitochondrial genes (mt-), and genes ending with Rik, as implemented in the filtering step of the analysis pipeline. For each gene that passed filtering, the log2 fold change (log2FC) values from the three comparisons (D1_vs_D0, D3_vs_D0, and D7_vs_D0) were averaged to obtain a mean log2FC representing the overall transcriptional trend. Genes were then separated into upregulated (mean log2FC > 0) and downregulated (mean log2FC < 0) groups and ranked within each direction based on the magnitude of the averaged log2FC. For visualization, we selected the top 30 upregulated and top 30 downregulated genes from each cell population for visualization. These ranked gene sets were used for producing the comparative heatmaps displayed in the main and Supplementary Materials figures.

### 2.11. Cell–Cell Communication Analysis

CellChat(v2.1.0) (https://github.com/jinworks/CellChat, accessed on 3 June 2026) is utilized in the R programming environment for quantitatively characterizing and comparing the inferred cell–cell communication networks using an integrated approach by combining social network analysis, pattern recognition, and manifold learning approaches. A database of known cytokine/chemokine receptor and ligand pairs was constructed using combined information from CellTalkDB v2 (https://deepwiki.com/jinworks/SpatialCellChat/2.2-cellchatdb:-ligand-receptor-interaction-database, accessed on 3 June 2026), which contains ~3300 validated molecular interactions, including ~40% of secrete autocrine/paracrine signaling interactions, ~17% of extracellular matrix (ECM)-receptor interactions, ~13% of cell–cell contact interactions and ~30% non-protein signaling; here we just included secreted signaling, ECM-receptor, cell–cell contact and non-protein signaling. To ensure the analysis’s relevance and accuracy, only ligand–receptor pairs exhibiting a *p*-value less than 0.05 were considered, allowing for a focused evaluation of the intricate relationships between diverse cell types.

### 2.12. Transcription Factor Regulatory Network Analysis

pySCENIC (version 0.11.2) (https://github.com/aertslab/pySCENIC, accessed on 3 June 2026), a computational approach for predicting critical regulators and identifying cell states from scRNA-seq data, is conducted for accessing the transcription factor (TF) regulons among all sub-cell types of RM and PTC cells across five-time experimental points. We filtered out genes expressed in fewer than 1% of cells and only kept the top 100 TFs with *p*-value < 0.005. TFannotations were obtained from the cisTarget database (https://github.com/aertslab/create_cisTarget_databases, accessed on 3 June 2026). This approach provided insights into the regulatory mechanisms driving cellular states.

### 2.13. Statistical Analysis

Data were presented as the mean ± standard error of the mean (s.e.m.) to summarize central tendency and variability. For statistical comparisons between multiple experimental groups, a one-way analysis of variance (ANOVA) was employed. In instances where experimental groups were compared over time or across multiple conditions, a two-way ANOVA was performed. For pairwise comparisons between two groups, an unpaired Student’s *t*-test was used to evaluate the significance of differences in group means. The unpaired version of the *t*-test was chosen as data were assumed to be independent between the two groups. A threshold for statistical significance was set at *p* < 0.05. Values that did not reach this threshold were considered not statistically significant and marked as “n.s.” to indicate *p* > 0.05. All statistical analyses were conducted using appropriate software, such as GraphPad Prism 11 and or R 4.5.1, to ensure robust and reproducible results.

## 3. Results

### 3.1. ScRNA-Seq Reveals Rapid Renal Macrophage Ablation, Regeneration, and Coordinated Kidney-Wide Transcriptional Remodeling

**Figure 1:**
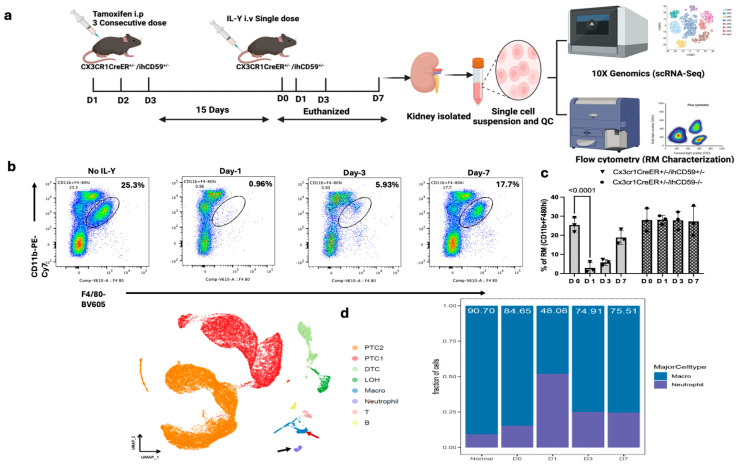
Flow cytometry and single-cell RNA sequencing confirmation of renal macrophage ablation and regeneration in mice. (**a**) Experimental design for scRNA-seq: Schematic representation of renal macrophage (RM) labeling with hCD59 via intraperitoneal administration of tamoxifen, followed by macrophage ablation using ILY. Kidneys from Cx3cr1CreER^+/−^/ihCD59^+/−^ mice were collected for single-cell RNA sequencing (scRNA-seq) using the 10× Genomics Chromium platform and for flow cytometric analysis. (**b**) Flow cytometric analysis of RMs: Representative plots depicting the proportion of RMs (CD11b^+^F4/80hi) at days 1, 3, and 7 following ILY administration (120 ng/g body weight), compared with control mice (no ILY treatment). (**c**) Ablation and regeneration of kidney-resident macrophages (RM): The figure illustrates the proportion of RM cells at different time points (D0, D1, D3, and D7) following intravenous ILY administration (120 ng/g body weight) in *Cx3cr1*CreER^+/−^/*ihCD59*^+/−^ and *Cx3cr1*CreER^+/−^/*ihCD59*^−/−^ mice, compared with control samples (D0, without ILY treatment). (**d**) UMAP visualization of scRNA-seq data identified distinct kidney cell populations, including renal macrophages. A stacked bar plot illustrates the proportion of macrophages among the myeloid cells (macrophages—red arrow + neutrophils—black arrow), which decreased from 84.65% at day 0 to 48.08% at day 1, followed by recovery to 74.91% at day 3 and 75.51% at day 7. For flow cytometry analyses, *n* = 3 mice per group at each time point. Data are presented as mean ± standard deviation (SD). Statistical significance was determined using two-way ANOVA, with *p* < 0.05 considered statistically significant. For single-cell RNA sequencing (scRNA-seq), *n* = 1 mouse per group at each time point. (**a**) was created using BioRender.com.

To effectively and swiftly target the RM while minimizing unintended effects on other tissues, we followed our previously established method [5,6] to administer ILY 15 days post-tamoxifen induction in *Cx3cr1CreER^+/−^/ihCD59^+/−^* mice (Figure 1a) [5,6]. By flow cytometry analysis (Figure 1b), we found that the depletion of RMs was effectively and specifically achieved after one day in ILY-injected *Cx3cr1CreER^+/−^/ihCD59^+/−^* mice but not vehicle-injected *Cx3cr1CreER^+/−^/ihCD59^+/−^* mice (Figure 1b,c). The population begins to replenish by day 3 post-injection, reaching approximately 25% of its initial level and by day 7 reaching approximately 75% (Figure 1b). This result is consistent with our previous finding that we can specifically manipulate RMs by rapidly ablating RMs and monitoring their regeneration in mice [5,6]. Then, we conducted scRNA-seq on ILY-treated and control *Cx3cr1CreER^+/−^/ihCD59^+/−^* mouse kidneys across baseline and days 1, 3, and 7 after RM ablation to define cellular and molecular responses (Figure 1a). Analysis of scRNA-seq data identified distinct clusters corresponding to major kidney epithelial populations, including PTC1 and PTC2, as well as immune cells such as macrophages and neutrophils (Figure 1d and Figure S1). Quantitative analysis of cellular proportions demonstrated a rapid and transient depletion of the macrophage-enriched compartment following injury. At baseline (day 0), the percentage of macrophage populations comprised 84.65% of the total analyzed myeloid cells (macrophages + neutrophils). This proportion sharply declined to 48.08% at day 1, indicating an acute disruption of the renal myeloid niche during the early injury phase. Notably, this reduction was followed by a progressive restoration of the macrophage compartment, recovering to 74.91% by day 3 and stabilizing at 75.51% by day 7. (Figure 1d and Figure S1b,c). This result is comparable with our finding observed by the flow cytometry (Figure 1b), further demonstrating the feasibility of the utilization of scRNA-seq for monitoring the RM changes after emptying the niche of RMs.

**Figure 2:**
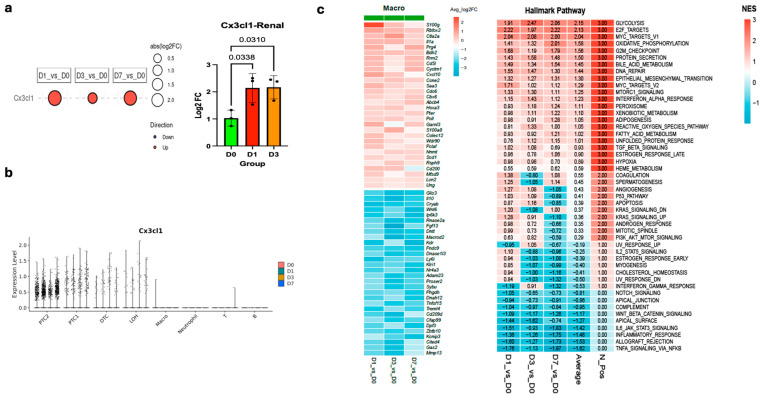
Renal epithelial cells drive Cx3cl1 upregulation after macrophage depletion and single-cell profiling reveals renal macrophage gene expression dynamics and pathway signatures across time. (**a**) RT-qPCR of whole kidney shows significant induction of *Cx3cl1* mRNA at days 1 and 3 after renal macrophage (RM) ablation; data are shown as fold change normalized to Beta-Actin (mean ± SD; *n* = 3 per time point per group, each sample run in duplicate; one-way ANOVA, *p* < 0.05). scRNA-seq dot plots confirm sustained *Cx3cl1* upregulation through day 7, indicating prolonged chemokine activation. (**b**) Cell-type-resolved scRNA-seq shows that multiple renal epithelial populations (PTC1, PTC2, LOH, DTC) maintain high Cx3cl1 expression at all post-ablation time points, identifying epithelial cells as the primary source of Cx3cl1 following RM niche depletion. (**c**) The first heatmap displays the top 30 upregulated and top 30 downregulated macrophage consistent differential expression genes ranked by mean log_2_ fold change across D1 vs. D0, D3 vs. D0, and D7 vs. D0, revealing consistent transcriptional responses to ILY treatment. Using these conserved DEGs, hallmark enrichment analysis was performed; a second heatmap summarizes the top 48 Hallmark pathways showing coordinated upregulated (red) and downregulated (blue) trends across the three comparisons.

Previously, we demonstrated that Cx3cl1 levels in serum and kidney significantly increased from day 1 to day 7 and gradually declined close to the baseline at day 7 post-rapid RM depletion [5]. This increase most likely results from the RM ablated kidney [5]. CX3CL1 is mainly produced by glomerular endothelial cells and the tubular epithelium and can be detected in many other cells, such as podocytes, mesangial cells, and stromal cells [5,37]. However, the exact cell population in the kidney responsible for producing Cx3cl1 remains unclear. Our current RT-qPCR analysis revealed marked upregulation of Cx3cl1 mRNA at days 1 and 3 following ablation (Figure 2a). To interrogate this process at single-cell resolution, we performed global scRNA-seq mapping, which not only corroborated these findings but also uncovered a sustained and progressive induction of Cx3cl1 across days 1, 3, and 7, indicating a prolonged chemotactic program activated in response to macrophage loss (Figure 2a). The Cx3cl1-positive cell population is primarily composed of multiple renal epithelial cell types, including PTC1, PTC2, DTC, and LOH in our analysis (Figure 2b). Notably, Cx3cl1 expression levels were comparable between kidneys from wild-type C57BL/6 (Normal) mice and day 0 Cx3cr1CreER^+/−^/ihCD59^+/−^ mice (S2a), demonstrating that basal renal Cx3cl1 expression is not affected by the genetic model before macrophage ablation. Together, these data confirm the previous finding at transcription level that *Cx3cl1* expression is increased after emptying RM niche and further demonstrate that renal epithelial cells, such as the PTC-1 and PTC-2, mainly produced Cx3cl1, a critical niche signaling in RM maintenance and regeneration.

To further evaluate the rigor of our scRNA-seq dataset, we performed global and single-cell transcriptomic analyses at baseline (D0) and from day 1 to day 7 post-ablation (D1–D7). We focused on the Macro, PTC1, PTC2, and Cx3cr1^+^ cell compartments and selected the top 30 upregulated (red) and top 30 downregulated (blue) differentially expressed genes (DEGs). Genes were ranked by the mean log_2_ fold change (log_2_FC) across the three comparisons (D1 vs. D0, D3 vs. D0, and D7 vs. D0) (Figure 2c and Figure S2b). Macrophages robustly upregulated inflammatory/stress-associated and proliferative programs (e.g., *S100a8*, *Lcn2*, *Cxcl10*, *Saa3*, *Rrm2*, *Cdc6*, *Pclaf*) while downregulating regulatory and angiogenic-associated signals (e.g., *Il10*, *Nr4a3*, *Cryab*, *Kdr*), consistent with the emergence of an injury-associated, repopulating macrophage state (Figure 2c, left panel). Collectively, these patterns suggest that replenishing macrophages in the post-ablation kidney niche adopt a more tissue-repairing, metabolically specialized, and microenvironment-modulating phenotype rather than a purely cytotoxic immune program. Hallmark pathway analysis of macrophages’ consistently upregulated genes further supported this interpretation (Figure 2c, right panel). Across D1, D3, and D7, macrophage signatures showed reproducible enrichment of metabolic and proliferative pathways, including Glycolysis, Oxidative Phosphorylation, E2F Targets, MYC Targets (V1/V2), G2M Checkpoint, and DNA Repair, together with stress-adaptation programs such as Unfolded Protein Response, Reactive Oxygen Species, and hypoxia (Figure 2c, right panel). In contrast, several canonical inflammatory/immune activation modules were relatively reduced including TNF-α-signaling via NF-κB, IL6-JAK-STAT3 signaling and inflammatory response, aligning with a shift toward reparative remodeling rather than overt inflammatory activation. The alignment of DEG-level changes with pathway enrichment across time highlights the internal consistency and biological relevance of macrophage responses captured by our scRNA-seq experiments, while the agreement between bulk and single-cell signatures across macrophages, tubular cells, and Cx3cr1^+^ populations underscore the rigor and accuracy of our approach.

### 3.2. Major CellCell Communication Dynamics Following RM Ablation

**Figure 3:**
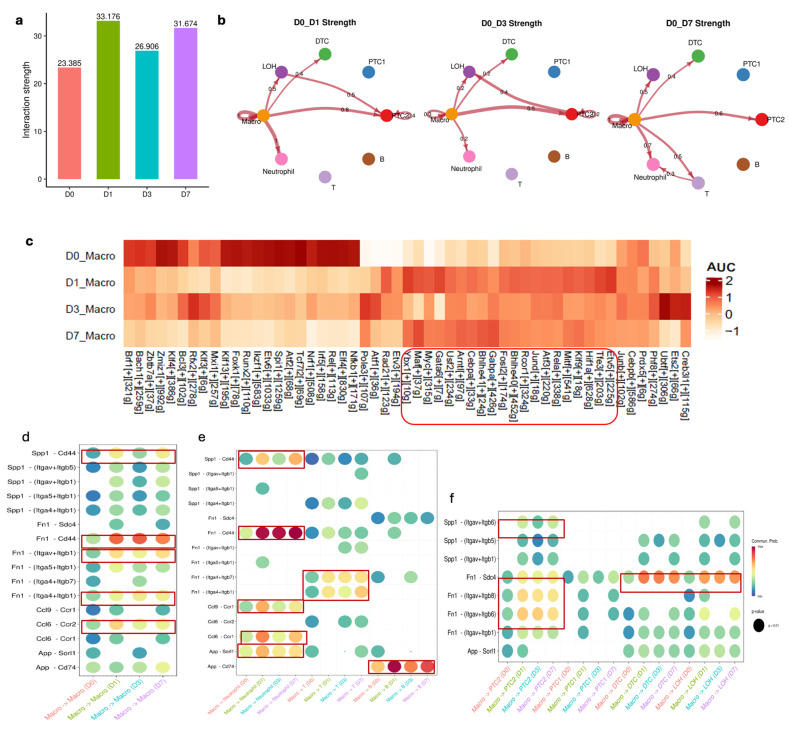
Cell-cell communication dynamics during renal macrophage (RM) depletion and regeneration. (**a**) Bar plot showing the total interaction strength of inferred cell-cell communication networks among eight major renal cell types across four time points. (**b**) Circular plots illustrating differential interaction strengths relative to D0 using the CellChat mouse database. Interactions include secreted signaling, ECM-receptor interactions, and direct cell-cell contacts. Edge width indicates interaction strength; red and blue denote increased and decreased signaling, respectively. Only the top 10% of interactions are shown. (**c**) Heatmap of transcription factors (TFs) predicted by SCENIC analysis in RMs across D0, D1, D3, and D7. Eighteen TFs (boxed), including *Etv5*, *Tfe3*, *Hif1a*, *Mitf*, *Rela*, *Atf3*, *Jund*, *Bhlhe40*, *Bhlhe41*, *Cebpa*, *Arntl*, *Klf9*, *Rcor1*, *Fosl2*, *Gabpa*, *Maf*, *Myc*, and *Ybx1*, exhibited sustained upregulation during RM regeneration. (**d**) Dot plot of autocrine signaling pathways within RMs. *Fn1-Cd44* showed the highest interaction probability, while *Spp1-Cd44* remained strongly active at D1, D3, and D7. (**e**) Dot plot of RM-to-immune cell signaling. RM-neutrophil interactions were dominated by *Fn1-Cd44*, *Spp1-Cd44*, and *Ccl9-Ccr1*; RM-B cell communication by *App-Cd74*; and RM-T cell interactions by *Fn1-(Itga4+Itgb1)* and *Fn1-(Itga4 + Itgb7)*. (**f**) Dot plot of RM-to-epithelial cell signaling. Major ligand–receptor pairs included *Fn1-Sdc4* across all time points and *Fn1-(Itgav + Itgb6)* and *Fn1-(Itgav + Itgb8)* in RM-PTC2 interactions. PTC1 exhibited relatively limited responsiveness.

To characterize intercellular signaling changes after RM ablation, we applied CellChat across eight major kidney cell types [17,38]. Global communication peaked at D1, decreased at D3, and partially recovered by D7, whereas D0 showed minimal signaling (Figure 3a). Circle plots of the top 10% differential interactions revealed strengthened crosstalk among RMs, epithelial cells (PTC2, LOH, DTC), and immune cells (neutrophils, T cells) after ablation (Figure 3b). To investigate the transcriptional programs underlying RM dynamics after ablation, we performed Transcription Factor Regulatory Network Analysis— “SCENIC” on RM scRNA-seq data across five time points (Normal, D0, D1, D3, D7) (Figure 3c). SCENIC identified 18 TF regulons with increased activity after ablation; however, focusing on TFs most relevant to myeloid cell differentiation, we highlighted a core subset that was consistently upregulated at D1, D3, and D7: *Tfe3*, *Mitf*, *Hif1a*, *Myc*, *Gabpa*, and *Rcor1* (Figure 3c). This pattern suggests that post-ablation RM restoration is driven by a differentiation-linked regulatory module rather than baseline homeostatic programs.

To connect these TFs with macrophage-centered communication programs, we used STRING to integrate the upregulated TFs with the ligands identified by CellChat (*Spp1*, *Fn1*, *App*, *Ccl6*, *Ccl9*) (S3a). STRING revealed significant network connectivity (PPI enrichment *p* = 7.66 × 10^−12^) and highlighted biological processes enriched in this integrated network, including interleukin-1-associated responses and myeloid differentiation-related programs (S3b). Especially *Myc*, *Hif1a*, *Mitf* and *Tfe3*, these three TFs are enriched in regulation of myeloid cell differentiation. Together, these data support a model in which a Tfe3/Mitf/Hif1a/Myc/Gabpa/Rcor1-centered transcriptional network coordinates macrophage regeneration and niche reconstitution by coupling differentiation programs with key signaling pathways active during renal repair.

The most dynamic pathways were Spp1, Fn1, and Ccl, with App emerging at D7. Fn1 signaling (notably Fn1-Cd44 and Fn1-Sdc4, plus integrin interactions) mediated RM-RM, RM–immune, and RM–epithelial communication (Figure 3d–f), while Spp1-Cd44 showed a transient dip at D3. Late-stage signaling featured App-Cd74 and App-Sorl1, and early chemokine recruitment was driven by Ccl6-Ccr2 and Ccl9-Ccr1. Overall, residual RMs rapidly reestablished themselves as communication hubs, coordinating epithelial and immune responses through temporally regulated Fn1, Spp1, and Ccl mediated networks.

### 3.3. Spp1 Signaling Promotes RM Regeneration Following Clodronate Induced Ablation

**Figure 4:**
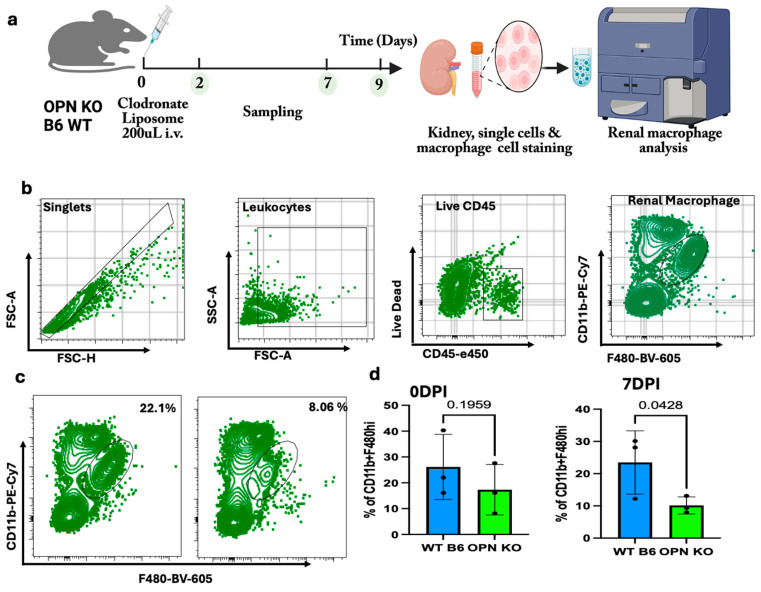
Blocking Spp1-CD44 signaling impairs renal macrophage regeneration after Clodronate-induced ablation: (**a**) Illustration of the experimental design for ablation and regeneration of renal macrophage in B6-WT and OPN KO mice using Clodronate liposomes. (**b**) Representative flow cytometry gating strategy for identifying renal macrophages defined as CD45^+^CD11b^+^F4/80hi cells isolated from kidney single-cell suspensions. Sequential gating excluded doublets and dead cells before macrophage subset identification. (**c**) Ablation of renal macrophages following intraperitoneal administration of 200 μL clodronate liposomes (CL) in C57BL/6 mice. Flow cytometric quantification at 48 h post-injection (2 DPI) demonstrated a ~65% reduction in CD45^+^CD11b^+^F4/80hi renal macrophages relative to PBS controls, confirming effective depletion of resident macrophage populations. (**d**) Regeneration kinetics of renal macrophages following CL-mediated depletion in B6-WT and OPN knockout (OPN KO) mice. A single intraperitoneal dose of CL (200 μL) was administered, and macrophage recovery was analyzed at 7 days (7 DPI) post-ablation. Data are presented as mean ± SEM (*n* = 3 mice per group). Statistical significance was determined using an unpaired one-tailed Student’s *t*-test. *p* < 0.05.

To define mechanisms underlying macrophage-mediated communication during renal repair, we focused on the Spp1 (osteopontin, OPN) signaling axis and tested its role in RM regeneration using Opn^−/−^ (*Spp1^−/−^*) mice. RMs were depleted by a single injection of clodronate liposomes (CL). In C57BL/6 (B6) mice, flow cytometry at 2 days post-injection (2 DPI) showed an ~65% reduction in CD45^+^CD11b^+^F4/80hi macrophages compared with PBS controls, confirming efficient RM ablation (Figure 4a–c). To assess Spp1-Cd44 signaling in RM repopulation, B6-WT and Opn^−/−^ mice underwent the same CL depletion, and recovery was quantified at 7 DPI and 9 DPI. WT mice rapidly regenerated RMs, returning to near-baseline levels by 7 DPI and remaining stable thereafter. In contrast, Opn^−/−^ mice exhibited a markedly impaired recovery trajectory, characterized by persistently reduced macrophage frequencies throughout the observation period. Notably, even at 7 days post-injury (DPI), macrophage levels remained significantly diminished compared to wild-type controls (Figure 4d). Together, these data identify Spp1/Opn signaling as a critical driver of RM regeneration and renal immune niche reconstitution following depletion.

### 3.4. Dynamic Remodeling of Renal Macrophage Subsets and Cell–Cell Communication Following Macrophage Ablation

**Figure 5:**
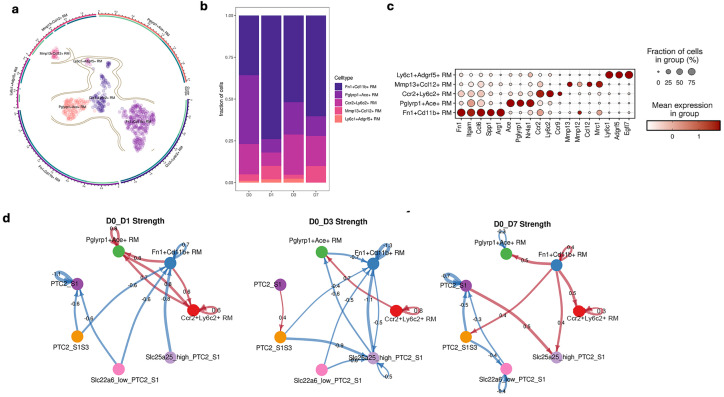
Visualization of subtypes of kidney renal macrophage (RM) and cell–cell communication among selected sub-cell types of RM and PTC2 in the kidney across four experiment time points. (**a**) UMAP embeddings of 594 renal macrophages (RM) from the mouse kidney across four experimental time points were analyzed following rigorous quality control and correction for batch effects. Unsupervised clustering and differential gene expression analysis identified four distinct RM subtypes. (**b**) A stacked bar plot illustrates the distribution of RM subtypes across the five experimental time points, highlighting their dynamic changes. (**c**) Dot plot of the top differentially expressed genes and known cell markers for RM subtypes. (**d**) Circular plots depicting the differential interaction strengths in the cell–cell communication network for seven selected sub-cell types of RM and PTC2, utilizing the CellChat mouse database. These comparisons are made between each time point, D1, D3, and D7, with D0. Interactions encompass secreted signaling, ECM-receptor interactions, and direct cell–cell contacts. The width of the edges represents interaction strength, while red (indicating increased signaling) and blue (indicating decreased signaling) edges highlight changes relative to D0. The plots display the top 25% of interaction strengths.

We next profiled renal macrophage (RM) dynamics following targeted ablation to characterize the processes governing tissue repopulation and niche restoration. Following efficient depletion at day 1 post-injury, the macrophage compartment at later time points primarily comprised newly recruited and differentiating cells, revealing increased cellular heterogeneity relative to homeostasis (day 0). Targeted sub-clustering across four time points identified five transcriptionally distinct RM subsets (Figure 5a–c) Among RMs, Fn1^+^Cd11b^+^ cells expressed Fn1, Itgam, Ccl6, Spp1, and Arg1, consistent with profibrotic tissue-remodeling “builder” macrophages [39,40,41]. Pglyrp1^+^Ace^+^ cells expressed Ace, Pglyrp1, and Nr4a1, suggesting antimicrobial and regulatory functions [42,43,44]. Ccr2^+^Ly6c2^+^ cells were enriched for recruited monocyte-derived markers (Ccr2, Ly6c2, Ccr9) and pro-inflammatory activity [45,46]. Mmp13^+^Ccl12^+^ cells expressed Mmp13/Mmp12, Ccl12, and Mrc1, indicating ECM remodeling and chemotaxis, while Ly6c1^+^Adgrf5^+^ cells (Ly6c1, Adgrf5, Egfl7) resembled niche-surveillance “sensor/guardian” macrophages. Temporally, Fn1^+^Cd11b^+^ and Mmp13^+^Ccl12^+^ peaked at day 1 and declined by days 3–7, whereas Pglyrp1^+^Ace^+^ and Ccr2^+^Ly6c2^+^ were reduced at day 1 but expanded later, approaching baseline by day 7 (Figure 5b), consistent with monocyte-driven replenishment and repair-associated transitions [39].

These results show that RM ablation induces dynamic remodeling in both macrophages and epithelial cells. Five RM subsets display distinct transcriptional programs-profibrotic, regulatory, inflammatory, remodeling, and surveillance with shifting proportions during injury and repair. Concurrently, PTC subclusters undergo transient loss and recovery, exhibiting stress adaptation, metabolic, and cytoskeletal changes. The emergence of two PTC populations (S1) suggests PTC1 represents injured epithelium with lower transporter expression and higher inflammatory signaling driven by immune–epithelial cross-talk.

At the major cell level, macrophages remained the primary signal senders after RM ablation, coordinating epithelial crosstalk, autoregulatory loops, and macrophage recruitment. PTC2 was a major recipient of RM-derived signals and showed self-regulation. To test whether subtype-level interactions differed, we focused on seven populations: Fn1^+^Cd11b^+^ RM, Pglyrp1^+^Ace^+^ RM, Ccr2^+^Ly6c2^+^ RM, and four PTC2 subtypes (PTC2_S1, PTC2_S1S3, Slc22a6_low_PTC2_S1, Slc25a25_high_PTC2_S1) (Figure 5d). Circle plots of the top 10% differential interactions revealed strong temporal shifts. At day 1, residual RMs showed increased communication compared with day 0, with Fn1^+^Cd11b^+^ RM as the dominant sender; Pglyrp1^+^Ace^+^ and Ccr2^+^Ly6c2^+^ RMs acted as both senders and receivers with autoregulation. At day 3, Ccr2^+^Ly6c2^+^ RM became the main signaling hub, interacting strongly with itself and Pglyrp1^+^Ace^+^ RM. By day 7, Fn1^+^Cd11b^+^ RM again dominated, targeting both RM subsets and PTC2 subtypes (PTC2_S1S3 and Slc25a25_high_PTC2_S1), while Ccr2^+^Ly6c2^+^ RM retained autoregulatory signaling (Figure 5d). Fn1–integrin interactions peaked at day 3 from Ccr2^+^Ly6c2^+^ RM, and Sirpb signaling was consistently enriched in Pglyrp1^+^Ace^+^ RM, indicating a subtype-restricted program. Overall, RM–PTC2 communication follows a temporal hierarchy: Fn1^+^Cd11b^+^ RM leads early and late, whereas Ccr2^+^Ly6c2^+^ RM dominates mid-phase signaling, together coordinating niche restoration through Fn1-, Spp1-, Ccl-, and App-driven networks.

## 4. Discussion

In this study, we used scRNA-seq analyses to monitor RM dynamics, renal global and single-cell transcriptomic changes in the response to acutely empty RMs niche in ILY-injected hCD59 labeled RMs mice [5,6]. First, scRNA-seq faithfully recapitulated these dynamics, resolving all major renal cell lineages and confirming the specificity and reproducibility of RM loss and recovery in line with established kidney single-cell atlases [9,12,47]. Second, we confirm that Cx3cl1 is a pivotal epithelial-derived signal that triggers and sustains RM regeneration [5] and documents that Cx3cl1 is mainly produced in PTC1 and PTC2 after depletion. These results are consistent with the previous transcriptomic analysis by others showing that CX3CL1 is highly expressed by tubular epithelium in injured kidneys and drives monocyte/macrophage attraction and retention in both homeostasis and disease states, including acute injury and immune nephritis models [48,49]. The sustained epithelial release of CX3CL1 may further support macrophage survival and differentiation, as CX3CR1 signaling has been implicated in macrophage longevity and function in other tissues [50]. Third, data self-validation of global and single-cell transcriptomic comparisons at three different time points showed highly reproducible gene-expression changes in macrophages, PTC1 and PTC2 and pathway changes in macrophages (Figure 2c). Only Ly6i and Treml4, established markers of tissue-resident (yolk-sac-derived) RMs, were consistently downregulated across Macro, Cx3cr1^+^, and bulk datasets, confirming selective depletion of resident macrophages. Macrophages displayed induction of DAMP and proliferative genes, consistent with inflammatory monocyte repopulation, alongside sustained upregulation of Cxcl10, a key chemokine driving monocyte-derived macrophage recruitment in kidney injury [51,52]. Shared upregulation of S100g and Cd5l (AIM) across macrophage populations reflected conserved injury-responsive programs, with CD5L implicated in macrophage remodeling and renal fibrosis across multiple kidney disease models [53,54,55]. Proximal tubules diverged into a metabolically rewired PTC1 state and an immune-stressed PTC2 state, the latter marked by Ly6e and Pfkfb3, linking epithelial stress to immune-metabolic pathways known from inflammatory macrophages [56]. Collectively, these data highlight tight macrophage–epithelial interdependence and establish ILY-ihCD59-mediated ablation with integrated transcriptomics as a robust framework to study RM niche regulation and epithelial homeostasis. Transcriptomic analysis showed that regenerating RMs do not immediately return to a homeostatic state but instead transiently acquire an injury-adaptive program marked by heightened metabolic activity, proliferation, and stress responses. Enrichment of glycolysis, oxidative phosphorylation, MYC- and E2F-driven cell-cycle programs, and DNA repair pathways indicate that RM regeneration is metabolically demanding and tightly linked to proliferative control. In contrast, canonical inflammatory pathways, including TNFα–NF-κB and IL-6–JAK–STAT3 signaling, were relatively subdued, consistent with a reparative rather than pro-inflammatory macrophage phenotype. This profile aligns with prior studies demonstrating that tissue-repair macrophages depend on metabolic reprogramming and proliferative capacity to restore organ integrity [1,57,58], and with kidney-specific evidence linking macrophage metabolic states to epithelial recovery and fibrosis limitation [59,60]. Our findings extend this paradigm by defining a transcriptional program uniquely associated with macrophage niche reconstitution following acute depletion. Collectively, our findings highlight a spatiotemporally coordinated epithelial–immune axis that orchestrates macrophage niche regeneration, with implications for targeted modulation of chemokine signaling in renal repair and chronic kidney diseases.

Cell–cell communication analysis revealed that RM ablation triggers a dynamic, time-dependent rewiring of intercellular signaling networks. RM-epithelial and RM-immune interactions peaked at days 1 and 7 post-ablation, with a transient reduction at day 3, consistent with macrophages acting as signaling hubs that coordinate early inflammatory responses followed by reparative programs [1,61,62,63]. Ligand–receptor analyses identified Spp1-Cd44 and Fn1-Cd44 as dominant pathways, implicating macrophage-driven ECM remodeling, fibrosis, and epithelial–immune communication [64,65,66] Spp1 signaling peaked at day 7, paralleling expansion of Spp1^+^ macrophages linked to chronic kidney injury and fibrotic repair [67,68], while Fn1-Cd44/Sdc4 interactions supported immune retention and matrix remodeling [69,70]. Functional analyses revealed that SPP1 is essential for macrophage regeneration following depletion, while being largely dispensable for steady-state homeostasis. Specifically, *Spp1^−/−^* mice exhibited a pronounced delay in macrophage repopulation after clodronate-mediated ablation, indicating a critical role for SPP1 in injury-induced macrophage renewal. Consistent with these findings, SPP1 has been implicated as a key mediator of immune cell–vascular interactions, particularly under inflammatory and reparative conditions [71,72]. Although clodronate liposome administration provides a simple and quick model to validate RM regeneration in Opn^−/−^ mice, it possesses limitations compared to our specific hCD59-ILY system. Clodronate may exert broader, less specific systemic effects on extra-renal phagocytes and induce a distinct inflammatory baseline. Future studies utilizing targeted CD44 receptor blockade or local rescue with recombinant osteopontin will be essential to precisely dissect the spatiotemporal dynamics of this axis. Together, these findings support a model in which macrophage- and epithelial-derived SPP1 establishes CD44-dependent autocrine and paracrine loops that drive macrophage recruitment, survival, and niche restoration during kidney repair [73,74]. Single-cell transcription factors regulatory network analysis identified 18 TFs selectively activated at days 1, 3, and 7 following RM ablation, implicating them in niche regeneration rather than homeostatic maintenance. Integration of these TFs with ligand–receptor pathways (Spp1, Fn1, App, Ccl6, Ccl9) revealed strong protein–protein interaction enrichment and highlighted IL-1-responsive and differentiation-related programs, consistent with IL-1 signaling as a key regulator of macrophage activation and myeloid differentiation during injury and repair [9]. Prominent TFs, including Hif1α, Myc, Cebpα, Klf family members, and BHLHE40/41, are well established drivers of macrophage metabolism, proliferation, and reparative polarization under inflammatory and hypoxic conditions [75,76,77,78,79]. TF clustering further revealed functional modules governing metabolic–proliferative reprogramming, stress and inflammatory responses, and chemokine-mediated leukocyte recruitment. Together, these data support a conserved differentiation program underlying RM regeneration, in which TF-driven induction of ligands such as Spp1 sustains autocrine and paracrine signaling essential for macrophage niche reconstitution.

After RM ablation, most macrophages detected from day 1 onward represent newly recruited cells, resulting in increased heterogeneity compared with the baseline RM pool. Single-cell analysis identified five RM subsets and ten proximal tubule cell (PTC) subtypes across time points, revealing greater epithelial diversity than previously appreciated. Among RMs, Fn1^+^Cd11b^+^ cells displayed a profibrotic remodeling phenotype [39], while Pglyrp1^+^Ace^+^ and Ccr2^+^Ly6c2^+^ subsets reflected antimicrobial and inflammatory monocyte-derived states. Mmp13^+^Ccl12^+^ macrophages were associated with ECM remodeling, whereas Ly6c1^+^Adgrf5^+^ cells represented niche-surveillance populations. Temporally, remodeling subsets peaked early, followed by expansion of inflammatory monocyte-derived populations, consistent with stepwise monocyte recruitment and differentiation during repair [15,80].

PTC sub-clustering revealed ten distinct epithelial states, with three PTC1 subtypes transiently reduced at day 1 and recovering by days 3–7, consistent with macrophage-dependent epithelial repair mediated by CSF-1 [81]. These PTC1 subsets reflected stress responses, metabolic adaptation, and cytoskeletal remodeling. Together, these data demonstrate coordinated temporal remodeling of macrophage and epithelial compartments after RM ablation, extending prior studies of RM heterogeneity and highlighting macrophage-epithelial cross-talk as a central driver of kidney regeneration [82,83,84,85,86]. Subtype-resolved communication analysis further demonstrated that RM-PTC2 crosstalk follows a temporal hierarchy. Fn1^+^Cd11b^+^ macrophages dominated early and late signaling through Fn1-Cd44 and integrin pathways, consistent with fibronectin’s established role in macrophage retention and tissue remodeling [39]. In contrast, Ccr2^+^Ly6c2^+^ macrophages became the principal signaling hub at the mid-phase, coinciding with peak monocyte recruitment and differentiation. Pglyrp1^+^Ace^+^ macrophages engaged distinct pathways, including App–Cd74 and Sirpb signaling, suggesting immunomodulatory roles not captured at the major cell level, in line with emerging evidence for APP-related signaling in macrophage immune regulation [87]. These data align with the view of macrophages as regulators of repair via structured networks [88,89]. The shifting roles of RM subsets mirror established macrophage plasticity during renal injury [20,90]. Furthermore, the prominent FN1 and SPP1 axis signaling accords with emerging models of macrophage-epithelial communication [20]. Our findings reveal a specific communication hierarchy where RM subsets sequentially regulate PTC2 signaling to coordinate epithelial repair. This highlights RM regeneration as a dynamically orchestrated inter-lineage process.

## 5. Conclusions

Acute depletion of renal macrophages (RMs) initiates a highly coordinated regenerative program characterized by rapid reconstitution of the macrophage niche through the sequential recruitment and differentiation of discrete macrophage subsets, including Ccr2^+^Ly6c2^+^, Pglyrp1^+^Ace^+^, and Fn1^+^Cd11b^+^ populations. Integrated single-cell transcriptomic and cell–cell communication analyses identify post-ablation macrophages as dominant signaling hubs that engage in dynamic bidirectional crosstalk with immune and proximal tubule epithelial (PTE) cells via key ligand–receptor pathways such as Spp1-Cd44 and Fn1-integrin. Collectively, these findings establish renal macrophage repopulation as a tightly regulated, transcription factor-dependent and communication-driven process that synchronizes immune–epithelial networks to restore kidney niche integrity, thereby providing mechanistic insight into regenerative repair while highlighting therapeutic opportunities to enhance regeneration and limit fibrosis. To guide future investigator efforts in fully translating these mechanisms, implementing targeted functional strategies such as in vivo anti-CD44 antibody blockades or localized rescue protocols using recombinant osteopontin will serve as a vital next step to validate these precise receptor–ligand dynamics. Despite the mechanistic clarity afforded by murine depletion–repopulation models, the transcriptional regulation of macrophage–tubular epithelial interactions in human kidney disease remains poorly defined. In particular, how injury-induced transcriptional programs govern macrophage recruitment, fate specification, and niche re-establishment through evolving intercellular signaling networks, and how macrophage heterogeneity integrates with epithelial stress responses to promote adaptive repair versus maladaptive fibrosis, remain unresolved. Addressing these gaps will require future studies employing chronic injury models, metabolic perturbations, and integrative multi-omics approaches in human biopsies and organoid systems to evaluate conservation, biomarker relevance, and therapeutic tractability of macrophage–epithelial communication pathways in kidney disease.

## Data Availability

All data associated with this study are present in the paper or the Supplementary Materials. The data that support the findings of this study are available from the corresponding author upon reasonable request. The datasets generated and analyzed during the current study are available from the corresponding author and first author upon reasonable request. Wild-type C57BL/6 are available in the Jackson Laboratory (Bar Harbor, ME). The *ihCD59^+/+^* mice, previously generated and backcrossed onto a C57BL/6 genetic background for at least seven generations is available in the Jackson Laboratory (Bar Harbor, ME). *Cx3cr1CreER^+/+^*, *ihCD59^+/+^* and ILY are available upon request through the material transfer agreements (MTA). The single-cell RNA sequencing (scRNA-seq) data generated in this study have been deposited in the Gene Expression Omnibus (GEO) repository under accession number GSE334728. The dataset is currently under embargo and will be made publicly available on 12 June 2026.

## Supplementary Materials

The following supporting information can be downloaded at https://www.mdpi.com/article/10.3390/cells15121102/s1, Figure S1: Landscape for cell compartments and sample construction of the single-cell RNA data of kidney tissues from mice model; Figure S2: (a) Baseline Cx3cl1 expression is unchanged in Cx3cr1CreER^+/−^/ihCD59^+/−^ kidneys prior to macrophage ablation; (b) Visualization of single-cell gene-expression dynamics across ILY-treated mouse kidneys; Figure S3: Renal Macrophage Transcription Factor Biological Function Prediction.

## References

[1] Wynn T.A., Vannella K.M. (2016). Macrophages in Tissue Repair, Regeneration, and Fibrosis. Immunity.

[2] Kratofil R.M., Kubes P., Deniset J.F. (2017). Monocyte Conversion During Inflammation and Injury. Arterioscler. Thromb. Vasc. Biol..

[3] Li L., Huang L., Sung S.S., Vergis A.L., Rosin D.L., Rose C.E., Lobo P.I., Okusa M.D. (2008). The chemokine receptors CCR2 and CX3CR1 mediate monocyte/macrophage trafficking in kidney ischemia-reperfusion injury. Kidney Int..

[4] Nelson P.J., Rees A.J., Griffin M.D., Hughes J., Kurts C., Duffield J. (2012). The renal mononuclear phagocytic system. J. Am. Soc. Nephrol..

[5] Liu F., Dai S., Feng D., Qin Z., Peng X., Sakamuri S., Ren M., Huang L., Cheng M., Mohammad K.E. (2020). Distinct fate, dynamics and niches of renal macrophages of bone marrow or embryonic origins. Nat. Commun..

[6] Islamuddin M., Evangelista J., Kabir M.E., Vidal A.K.N., Liu S., Qin X. (2025). Rapid Depletion of Renal Macrophages Using Human CD59/Intermedilysin Cell Ablation Tool. J. Vis. Exp..

[7] Lee S., Huen S., Nishio H., Nishio S., Lee H.K., Choi B.S., Ruhrberg C., Cantley L.G. (2011). Distinct macrophage phenotypes contribute to kidney injury and repair. J. Am. Soc. Nephrol..

[8] Wang W., Wang X., Chun J., Vilaysane A., Clark S., French G., Bracey N.A., Trpkov K., Bonni S., Duff H.J. (2013). Inflammasome-independent NLRP3 augments TGF-β signaling in kidney epithelium. J. Immunol..

[9] Chen Z., Li Y., Yuan Y., Lai K., Ye K., Lin Y., Lan R., Chen H., Xu Y. (2023). Single-cell sequencing reveals homogeneity and heterogeneity of the cytopathological mechanisms in different etiology-induced AKI. Cell Death Dis..

[10] Wang Y., Harris D.C. (2011). Macrophages in renal disease. J. Am. Soc. Nephrol..

[11] Cliff C.L., Squires P.E., Hills C.E. (2024). Tonabersat suppresses priming/activation of the NOD-like receptor protein-3 (NLRP3) inflammasome and decreases renal tubular epithelial-to-macrophage crosstalk in a model of diabetic kidney disease. Cell Commun. Signal.

[12] Conway B.R., O’Sullivan E.D., Cairns C., O’Sullivan J., Simpson D.J., Salzano A., Connor K., Ding P., Humphries D., Stewart K. (2020). Kidney Single-Cell Atlas Reveals Myeloid Heterogeneity in Progression and Regression of Kidney Disease. J. Am. Soc. Nephrol..

[13] Xu H., Chai H., Chen M., Zhu R., Jiang S., Liu X., Wang Y., Chen J., Wei J., Mao Y. (2024). Single-cell RNA sequencing identifies a subtype of FN1 + tumor-associated macrophages associated with glioma recurrence and as a biomarker for immunotherapy. Biomark. Res..

[14] Willenborg S., Lucas T., van Loo G., Knipper J.A., Krieg T., Haase I., Brachvogel B., Hammerschmidt M., Nagy A., Ferrara N. (2012). CCR2 recruits an inflammatory macrophage subpopulation critical for angiogenesis in tissue repair. Blood.

[15] Dick S.A., Wong A., Hamidzada H., Nejat S., Nechanitzky R., Vohra S., Mueller B., Zaman R., Kantores C., Aronoff L. (2022). Three tissue resident macrophage subsets coexist across organs with conserved origins and life cycles. Sci. Immunol..

[16] Qie C., Jiang J., Liu W., Hu X., Chen W., Xie X., Liu J. (2020). Single-cell RNA-Seq reveals the transcriptional landscape and heterogeneity of skin macrophages in Vsir^−/−^ murine psoriasis. Theranostics.

[17] Jin S., Plikus M.V., Nie Q. (2025). CellChat for systematic analysis of cell-cell communication from single-cell transcriptomics. Nat. Protoc..

[18] Jin X., Jian Z., Ma Y., Wen J., Chao N., Chen X., Xiang L., Yuan Y., Liu L., Li Y. (2025). Single-Cell RNA Sequencing Analysis Reveals the Role of Macrophage-Mediated CD44-AKT-CCL2 Pathways in Renal Tubule Injury during Calcium Oxalate Crystal Formation. Research.

[19] Niu D., Yang J.J., He D.F. (2025). The role of macrophages in renal fibrosis and therapeutic prospects. PeerJ.

[20] Zhang Y.L., Tang T.T., Wang B., Wen Y., Feng Y., Yin Q., Jiang W., Zhang Y., Li Z.L., Wu M. (2024). Identification of a Novel ECM Remodeling Macrophage Subset in AKI to CKD Transition by Integrative Spatial and Single-Cell Analysis. Adv. Sci..

[21] Wen Y., Lu X., Ren J., Privratsky J.R., Yang B., Rudemiller N.P., Zhang J., Griffiths R., Jain M.K., Nedospasov S.A. (2019). KLF4 in Macrophages Attenuates TNFα-Mediated Kidney Injury and Fibrosis. J. Am. Soc. Nephrol..

[22] Liao X., Sharma N., Kapadia F., Zhou G., Lu Y., Hong H., Paruchuri K., Mahabeleshwar G.H., Dalmas E., Venteclef N. (2011). Krüppel-like factor 4 regulates macrophage polarization. J. Clin. Investig..

[23] Xu H., Zhu J., Smith S., Foldi J., Zhao B., Chung A.Y., Outtz H., Kitajewski J., Shi C., Weber S. (2012). Notch-RBP-J signaling regulates the transcription factor IRF8 to promote inflammatory macrophage polarization. Nat. Immunol..

[24] Xiao H., Wu Y.P., Yang C.C., Yi Z., Zeng N., Xu Y., Zeng H., Deng P., Zhang Q., Wu M. (2020). Knockout of E2F1 enhances the polarization of M2 phenotype macrophages to accelerate the wound healing process. Kaohsiung J. Med. Sci..

[25] Zhang M.Z., Yao B., Yang S., Jiang L., Wang S., Fan X., Yin H., Wong K., Miyazawa T., Chen J. (2012). CSF-1 signaling mediates recovery from acute kidney injury. J. Clin. Investig..

[26] Menke J., Iwata Y., Rabacal W.A., Basu R., Yeung Y.G., Humphreys B.D., Wada T., Schwarting A., Stanley E.R., Kelley V.R. (2009). CSF-1 signals directly to renal tubular epithelial cells to mediate repair in mice. J. Clin. Investig..

[27] Trostel J., Truong L.D., Roncal-Jimenez C., Miyazaki M., Miyazaki-Anzai S., Kuwabara M., McMahan R., Andres-Hernando A., Sato Y., Jensen T. (2018). Different effects of global osteopontin and macrophage osteopontin in glomerular injury. Am. J. Physiol. Ren. Physiol..

[28] Duffield J.S., Forbes S.J., Constandinou C.M., Clay S., Partolina M., Vuthoori S., Wu S., Lang R., Iredale J.P. (2005). Selective depletion of macrophages reveals distinct, opposing roles during liver injury and repair. J. Clin. Investig..

[29] Hua L., Shi J., Shultz L.D., Ren G. (2018). Genetic Models of Macrophage Depletion. Methods Mol. Biol..

[30] Murray P.J., Wynn T.A. (2011). Protective and pathogenic functions of macrophage subsets. Nat. Rev. Immunol..

[31] Hu W., Ferris S.P., Tweten R.K., Wu G., Radaeva S., Gao B., Bronson R.T., Halperin J.A., Qin X. (2008). Rapid conditional targeted ablation of cells expressing human CD59 in transgenic mice by intermedilysin. Nat. Med..

[32] Feng D., Dai S., Liu F., Ohtake Y., Zhou Z., Wang H., Zhang Y., Kearns A., Peng X., Zhu F. (2016). Cre-inducible human CD59 mediates rapid cell ablation after intermedilysin administration. J. Clin. Investig..

[33] Giddings K.S., Zhao J., Sims P.J., Tweten R.K. (2004). Human CD59 is a receptor for the cholesterol-dependent cytolysin intermedilysin. Nat. Struct. Mol. Biol..

[34] Tsai Y.C., Kuo M.C., Huang J.C., Chang W.A., Wu L.Y., Huang Y.C., Chang C.Y., Lee S.C., Hsu Y.L. (2023). Single-cell transcriptomic profiles in the pathophysiology within the microenvironment of early diabetic kidney disease. Cell Death Dis..

[35] Miao Z., Balzer M.S., Ma Z., Liu H., Wu J., Shrestha R., Aranyi T., Kwan A., Kondo A., Pontoglio M. (2021). Single cell regulatory landscape of the mouse kidney highlights cellular differentiation programs and disease targets. Nat. Commun..

[36] Park J., Shrestha R., Qiu C., Kondo A., Huang S., Werth M., Li M., Barasch J., Suszták K. (2018). Single-cell transcriptomics of the mouse kidney reveals potential cellular targets of kidney disease. Science.

[37] Kim K.W., Vallon-Eberhard A., Zigmond E., Farache J., Shezen E., Shakhar G., Ludwig A., Lira S.A., Jung S. (2011). In vivo structure/function and expression analysis of the CX3C chemokine fractalkine. Blood.

[38] Jin S., Guerrero-Juarez C.F., Zhang L., Chang I., Ramos R., Kuan C.H., Myung P., Plikus M.V., Nie Q. (2021). Inference and analysis of cell-cell communication using CellChat. Nat. Commun..

[39] Ouyang Q., Wang C., Sang T., Tong Y., Zhang J., Chen Y., Wang X., Wu L., Wang X., Liu R. (2024). Depleting profibrotic macrophages using bioactivated in vivo assembly peptides ameliorates kidney fibrosis. Cell Mol. Immunol..

[40] Lou Y., Li P.H., Liu X.Q., Wang T.X., Liu Y.L., Chen C.C., Ma K.L. (2024). ITGAM-mediated macrophages contribute to basement membrane damage in diabetic nephropathy and atherosclerosis. BMC Nephrol..

[41] Liu J., Guo F., Chen X., Fu P., Ma L. (2024). Integrin αM promotes macrophage alternative M2 polarization in hyperuricemia-related chronic kidney disease. MedComm.

[42] Moratal C., Laurain A., Naïmi M., Florin T., Esnault V., Neels J.G., Chevalier N., Chinetti G., Favre G. (2021). Regulation of Monocytes/Macrophages by the Renin-Angiotensin System in Diabetic Nephropathy: State of the Art and Results of a Pilot Study. Int. J. Mol. Sci..

[43] Slonova D., Posvyatenko A., Kibardin A., Sysolyatina E., Lyssuk E., Ermolaeva S., Obydennyi S., Gnuchev N., Georgiev G., Severinov K. (2020). Human Short Peptidoglycan Recognition Protein PGLYRP1/Tag-7/PGRP-S Inhibits Listeria monocytogenes Intracellular Survival in Macrophages. Front. Cell Infect. Microbiol..

[44] Westbrook L., Johnson A.C., Regner K.R., Williams J.M., Mattson D.L., Kyle P.B., Henegar J.R., Garrett M.R. (2014). Genetic susceptibility and loss of Nr4a1 enhances macrophage-mediated renal injury in CKD. J. Am. Soc. Nephrol..

[45] Miyake K., Ito J., Takahashi K., Nakabayashi J., Brombacher F., Shichino S., Yoshikawa S., Miyake S., Karasuyama H. (2024). Single-cell transcriptomics identifies the differentiation trajectory from inflammatory monocytes to pro-resolving macrophages in a mouse skin allergy model. Nat. Commun..

[46] Shi C., Pamer E.G. (2011). Monocyte recruitment during infection and inflammation. Nat. Rev. Immunol..

[47] Yashchenko A., Bland S.J., Song C.J., Ahmed U.K.B., Sharp R., Darby I.G., Cordova A.M., Smith M.E., Lever J.M., Li Z. (2023). Cx3cr1 controls kidney resident macrophage heterogeneity. Front. Immunol..

[48] von Vietinghoff S., Kurts C. (2021). Regulation and function of CX3CR1 and its ligand CX3CL1 in kidney disease. Cell Tissue Res..

[49] Cormican S., Griffin M.D. (2021). Fractalkine (CX3CL1) and Its Receptor CX3CR1: A Promising Therapeutic Target in Chronic Kidney Disease?. Front. Immunol..

[50] Imai T., Yasuda N. (2016). Therapeutic intervention of inflammatory/immune diseases by inhibition of the fractalkine (CX3CL1)-CX3CR1 pathway. Inflamm. Regen..

[51] Petrovic-Djergovic D., Popovic M., Chittiprol S., Cortado H., Ransom R.F., Partida-Sánchez S. (2015). CXCL10 induces the recruitment of monocyte-derived macrophages into kidney, which aggravate puromycin aminonucleoside nephrosis. Clin. Exp. Immunol..

[52] Gao L., Zhong X., Jin J., Li J., Meng X.M. (2020). Potential targeted therapy and diagnosis based on novel insight into growth factors, receptors, and downstream effectors in acute kidney injury and acute kidney injury-chronic kidney disease progression. Signal Transduct. Target. Ther..

[53] Irabu H., Shimizu M., Kaneko S., Inoue N., Mizuta M., Tasaki Y., Ohta K., Yachie A., Wada T. (2021). Apoptosis inhibitor of macrophage as a biomarker for disease activity in Japanese children with IgA nephropathy and Henoch-Schönlein purpura nephritis. Pediatr. Res..

[54] Li H., Li D., Ledru N., Xuanyuan Q., Wu H., Asthana A., Byers L.N., Tullius S.G., Orlando G., Waikar S.S. (2024). Transcriptomic, epigenomic, and spatial metabolomic cell profiling redefines regional human kidney anatomy. Cell Metab..

[55] Chen Z., Chen C., Lai K., Wu C., Wu F., Chen Z., Ye K., Xie J., Ma H., Chen H. (2024). GSDMD and GSDME synergy in the transition of acute kidney injury to chronic kidney disease. Nephrol. Dial. Transplant..

[56] Kolliniati O., Ieronymaki E., Vergadi E., Tsatsanis C. (2022). Metabolic Regulation of Macrophage Activation. J. Innate Immun..

[57] Lavin Y., Winter D., Blecher-Gonen R., David E., Keren-Shaul H., Merad M., Jung S., Amit I. (2014). Tissue-resident macrophage enhancer landscapes are shaped by the local microenvironment. Cell.

[58] Lee S.H., Kim K.H., Lee S.M., Park S.J., Lee S., Cha R.H., Lee J.W., Kim D.K., Kim Y.S., Ye S.K. (2024). STAT3 blockade ameliorates LPS-induced kidney injury through macrophage-driven inflammation. Cell Commun. Signal.

[59] Kang H.M., Ahn S.H., Choi P., Ko Y.A., Han S.H., Chinga F., Park A.S., Tao J., Sharma K., Pullman J. (2015). Defective fatty acid oxidation in renal tubular epithelial cells has a key role in kidney fibrosis development. Nat. Med..

[60] Zhu Y., Tan J., Wang Y., Gong Y., Zhang X., Yuan Z., Lu X., Tang H., Zhang Z., Jiang X. (2024). Atg5 deficiency in macrophages protects against kidney fibrosis via the CCR6-CCL20 axis. Cell Commun. Signal.

[61] Watanabe S., Alexander M., Misharin A.V., Budinger G.R.S. (2019). The role of macrophages in the resolution of inflammation. J. Clin. Investig..

[62] Guenin-Mace L., Konieczny P., Naik S. (2023). Immune-Epithelial Cross Talk in Regeneration and Repair. Annu. Rev. Immunol..

[63] Lin Y., Yang Q., Zeng R. (2025). Crosstalk between macrophages and adjacent cells in AKI to CKD transition. Ren. Fail..

[64] Fei C., Chen Y., Tan R., Yang X., Wu G., Li C., Shi J., Le S., Yang W., Xu J. (2025). Single-cell multi-omics analysis identifies SPP1(+) macrophages as key drivers of ferroptosis-mediated fibrosis in ligamentum flavum hypertrophy. Biomark. Res..

[65] Chen X., Yang Y., Sun S., Liu Q., Yang Y., Jiang L. (2024). CX3C chemokine: Hallmarks of fibrosis and ageing. Pharmacol. Res..

[66] Tong W., Wang T., Bai Y., Yang X., Han P., Zhu L., Zhang Y., Shen Z. (2024). Spatial transcriptomics reveals tumor-derived SPP1 induces fibroblast chemotaxis and activation in the hepatocellular carcinoma microenvironment. J. Transl. Med..

[67] Sinha S.K., Mellody M., Carpio M.B., Damoiseaux R., Nicholas S.B. (2023). Osteopontin as a Biomarker in Chronic Kidney Disease. Biomedicines.

[68] Hoeft K., Schaefer G.J.L., Kim H., Schumacher D., Bleckwehl T., Long Q., Klinkhammer B.M., Peisker F., Koch L., Nagai J. (2023). Platelet-instructed SPP1(+) macrophages drive myofibroblast activation in fibrosis in a CXCL4-dependent manner. Cell Rep..

[69] Möckel T., Boegel S., Schwarting A. (2023). Transcriptome analysis of renal ischemia/reperfusion (I/R) injury in BAFF and BAFF-R deficient mice. PLoS ONE.

[70] Lei L., Xiang Y.X., Luo M.L., Zhang Z.Y., Wu H.W., Tang C., Cui T.J., Zhang X.M., Wang X.H., Delic D. (2025). Intercellular Communication Network of CellChat Uncovers Mechanisms of Kidney Fibrosis Based on Single-Cell RNA Sequencing. Kidney Blood Press. Res..

[71] Zhao Y., Huang Z., Gao L., Ma H., Chang R. (2024). Osteopontin/SPP1: A potential mediator between immune cells and vascular calcification. Front. Immunol..

[72] Khamissi F.Z., Ning L., Kefaloyianni E., Dun H., Arthanarisami A., Keller A., Atkinson J.J., Li W., Wong B., Dietmann S. (2022). Identification of kidney injury released circulating osteopontin as causal agent of respiratory failure. Sci. Adv..

[73] Rittling S.R. (2011). Osteopontin in macrophage function. Expert. Rev. Mol. Med..

[74] Ng C.W., Tam I.Y.S., Sam S.W., Yu Y., Lau H.Y.A. (2018). Immobilized Osteopontin Enhances Adhesion but Suppresses Cytokine Release of Anti-IgE Activated Human Mast Cells. Front. Immunol..

[75] Corcoran S.E., O’Neill L.A. (2016). HIF1α and metabolic reprogramming in inflammation. J. Clin. Investig..

[76] Hutami I.R., Izawa T., Khurel-Ochir T., Sakamaki T., Iwasa A., Tanaka E. (2021). Macrophage Motility in Wound Healing Is Regulated by HIF-1α via S1P Signaling. Int. J. Mol. Sci..

[77] Lee B., Qiao L., Lu M., Yoo H.S., Cheung W., Mak R., Schaack J., Feng G.S., Chi N.W., Olefsky J.M. (2014). C/EBPα regulates macrophage activation and systemic metabolism. Am. J. Physiol. Endocrinol. Metab..

[78] Zhang Y., Du C., Wang W., Qiao W., Li Y., Zhang Y., Sheng S., Zhou X., Zhang L., Fan H. (2024). Glucocorticoids increase adiposity by stimulating Krüppel-like factor 9 expression in macrophages. Nat. Commun..

[79] Xu S., Li H., Han J., Xu Y., Li N., Che W., Liu F., Yue W. (2025). Klf9 promotes the repair of myocardial infarction by regulating macrophage recruitment and polarization. JCI Insight.

[80] Gautier E.L., Jakubzick C., Randolph G.J. (2009). Regulation of the migration and survival of monocyte subsets by chemokine receptors and its relevance to atherosclerosis. Arterioscler. Thromb. Vasc. Biol..

[81] Alikhan M.A., Jones C.V., Williams T.M., Beckhouse A.G., Fletcher A.L., Kett M.M., Sakkal S., Samuel C.S., Ramsay R.G., Deane J.A. (2011). Colony-stimulating factor-1 promotes kidney growth and repair via alteration of macrophage responses. Am. J. Pathol..

[82] Chew C., Brand O.J., Yamamura T., Lawless C., Morais M., Zeef L., Lin I.H., Howell G., Lui S., Lausecker F. (2024). Kidney resident macrophages have distinct subsets and multifunctional roles. Matrix Biol..

[83] Ledru N., Wilson P.C., Muto Y., Yoshimura Y., Wu H., Li D., Asthana A., Tullius S.G., Waikar S.S., Orlando G. (2024). Predicting proximal tubule failed repair drivers through regularized regression analysis of single cell multiomic sequencing. Nat. Commun..

[84] Lake B.B., Menon R., Winfree S., Hu Q., Melo Ferreira R., Kalhor K., Barwinska D., Otto E.A., Ferkowicz M., Diep D. (2023). An atlas of healthy and injured cell states and niches in the human kidney. Nature.

[85] Abdelkarim G.E., Gertz K., Harms C., Katchanov J., Dirnagl U., Szabó C., Endres M. (2001). Protective effects of PJ34, a novel, potent inhibitor of poly(ADP-ribose) polymerase (PARP) in in vitro and in vivo models of stroke. Int. J. Mol. Med..

[86] Ma Y., Yang F., Yang J., Wang K., Hu J., Wu Q. (2025). The multifaceted role of macrophages in kidney physiology and diseases. Front. Immunol..

[87] Hickman S., Izzy S., Sen P., Morsett L., El Khoury J. (2018). Microglia in neurodegeneration. Nat. Neurosci..

[88] Ricardo S.D., van Goor H., Eddy A.A. (2008). Macrophage diversity in renal injury and repair. J. Clin. Investig..

[89] Guo C., Cui Y., Jiao M., Yao J., Zhao J., Tian Y., Dong J., Liao L. (2023). Crosstalk between proximal tubular epithelial cells and other interstitial cells in tubulointerstitial fibrosis after renal injury. Front. Endocrinol..

[90] Qu Z., Chu J., Jin S., Yang C., Zang J., Zhang J., Xu D., Cheng M. (2025). Tissue-resident macrophages and renal diseases: Landscapes and treatment directions. Front. Immunol..

