# Supplementary material for: The Reconstitution of the Macrophage Niche Reveals Dynamic Transcriptional and Renal Macrophage–Epithelial Communication Networks
Source: Cells. 2026 Jun 18;15(12):1102. doi: 10.3390/cells15121102 (PMC13297203; doi:10.3390/cells15121102)
Supplement: Supplementary file 1 [file cells-15-01102-s001.zip › cells-4299291-supplementary.pdf]

SUPPLEMENTARY MATERIALS

a

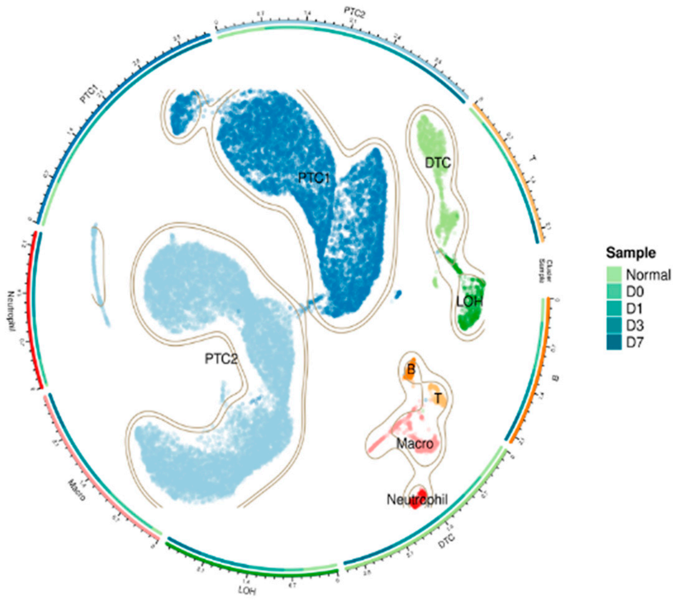

b

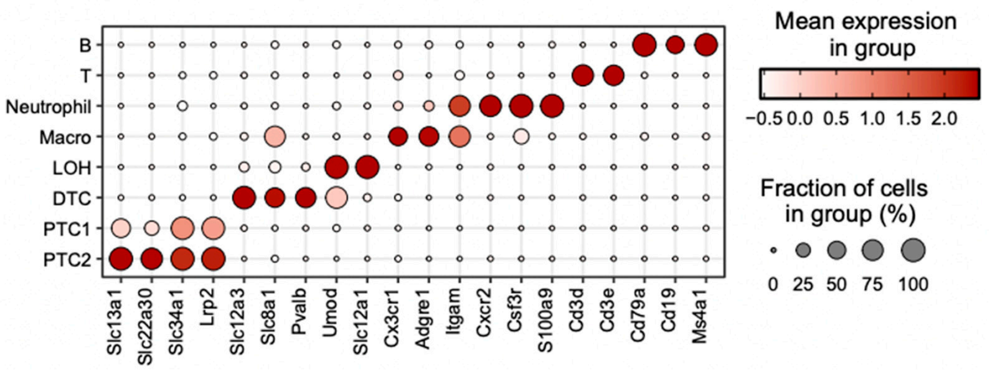

c

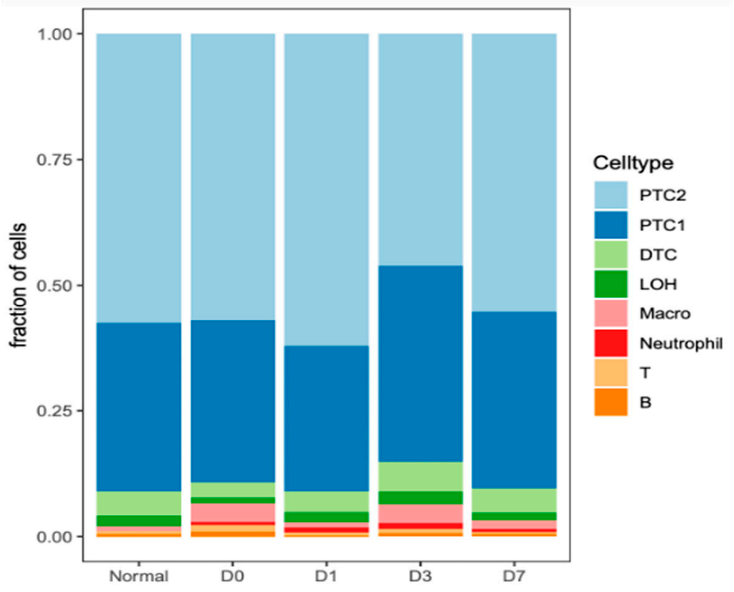

Supplementary Figure S1: Landscape for cell compartments and sample construction of the single-cell RNA data of kidney tissues from mice model: (a) Uniform manifold approximation and projection (UMAP) embeddings of total 27,396 mice renal cells from 5 time points after quality control and removal of batch effects. We identified total 7 major cell types and Proximal Tubular Cell (PTC) separate to PTC1 and PTC2 based on unsupervised clustering and mitochondrial percentage difference. The 2 color tracks in the circular plots (from outside to inside) indicate cell type and sample information. **(b)** Dot plot of top differentially expressed genes and known cell markers for each renal cell types. **(c)** Stacked bar plot showing the distribution of major cell types across 5 time points, percentage of renal macrophage dramatically decrease from D0 to D1 then gradually generate back from D1 to D7. Abbreviations: Proximal Tubular Cell (PTC), Distal Tubular Cell (DTC), Loop of Helene (LOH), Macrophage (Macro), T Cell (T), B Cell (B).

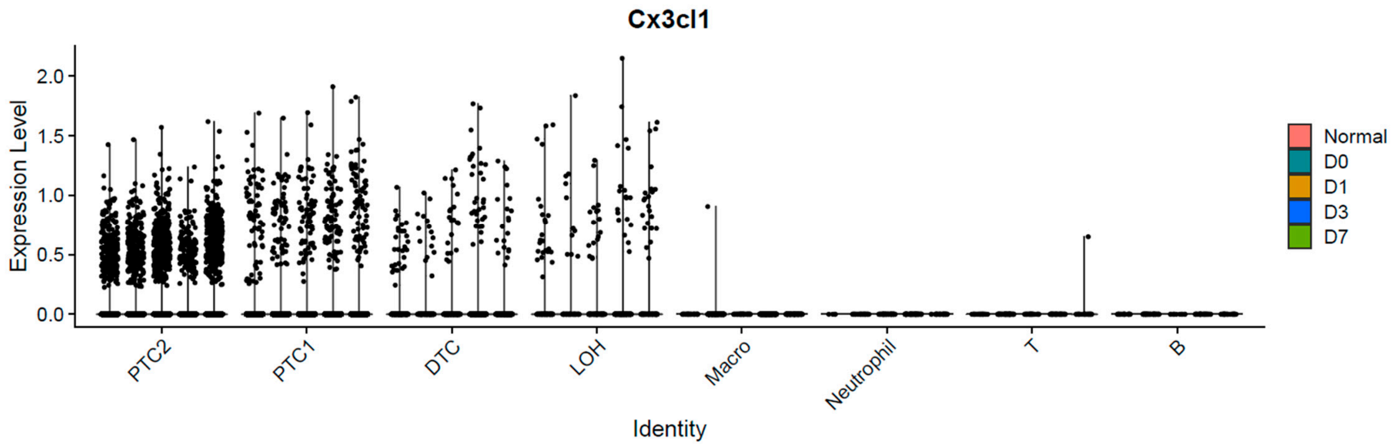

Supplementary Figure S2a. Baseline Cx3cl1 expression is unchanged in Cx3cr1CreER+/−/ihCD59+/− kidneys prior to macrophage ablation. Comparison of Cx3cl1 expression levels between kidneys from wild-type C57BL/6 mice and Day 0 Cx3cr1CreER+/−/ihCD59+/− mice revealed no significant differences, indicating that the genetic background and experimental setup do not alter basal renal Cx3cl1 expression prior to renal macrophage depletion.

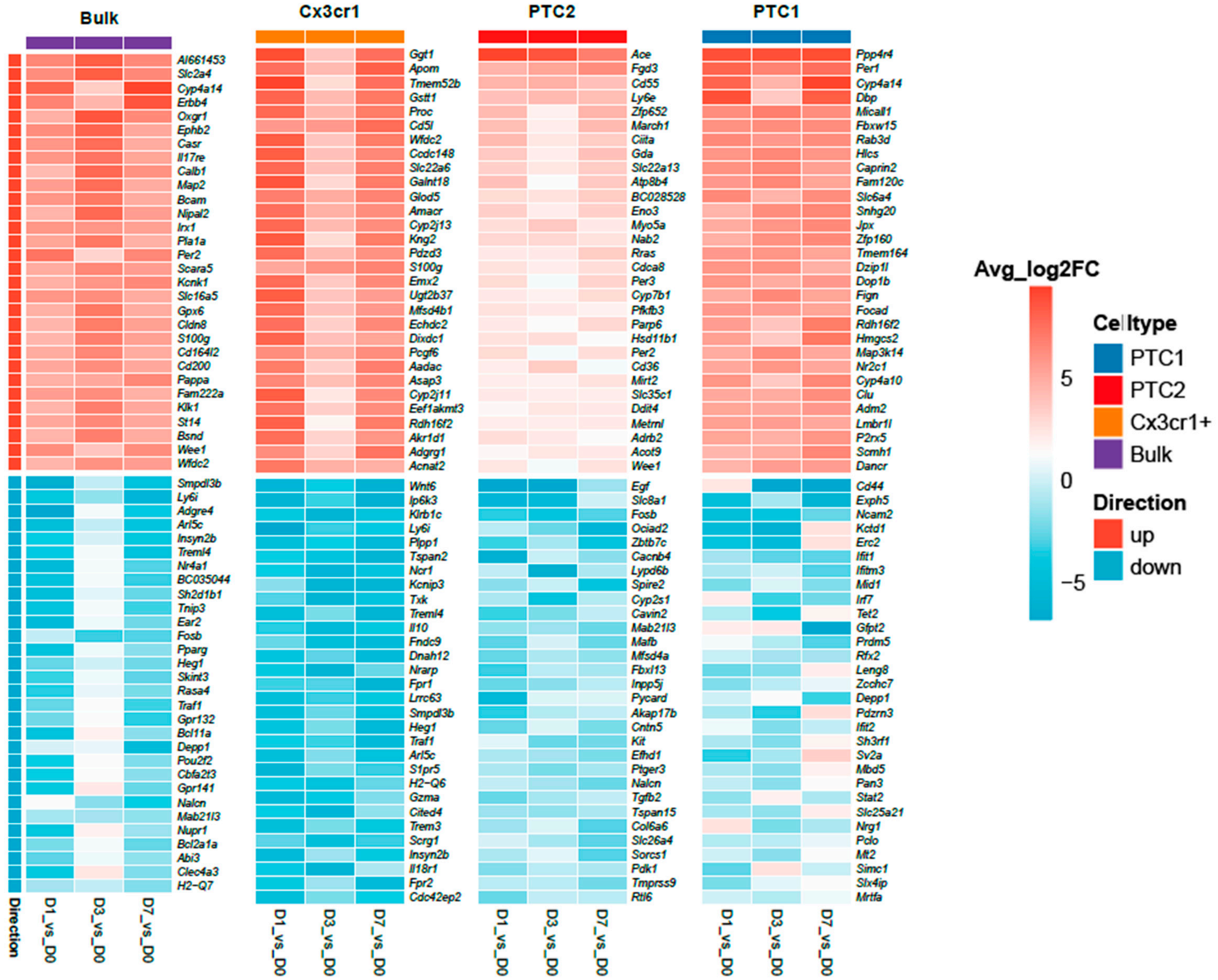

Supplementary Figure S2b: Visualization of single-cell gene-expression dynamics across ILY-treated mouse kidneys. Heatmap showing the top 30 upregulated (red) and top 30 downregulated (blue) differentially expressed genes (DEGs) identified in Bulk kidney cell populations, PTC1, PTC2, and Cx3cr1^{+} cell populations, profiled by scRNA-seq. Genes were ranked according to the mean $\log_2$ fold-change ($\log_2$FC) across the three time-point comparisons (D1 vs D0, D3 vs D0, and D7 vs D0). The heatmap illustrates consistent expression trends across groups, highlighting transcriptional responses to ILY treatment at the single-cell level.

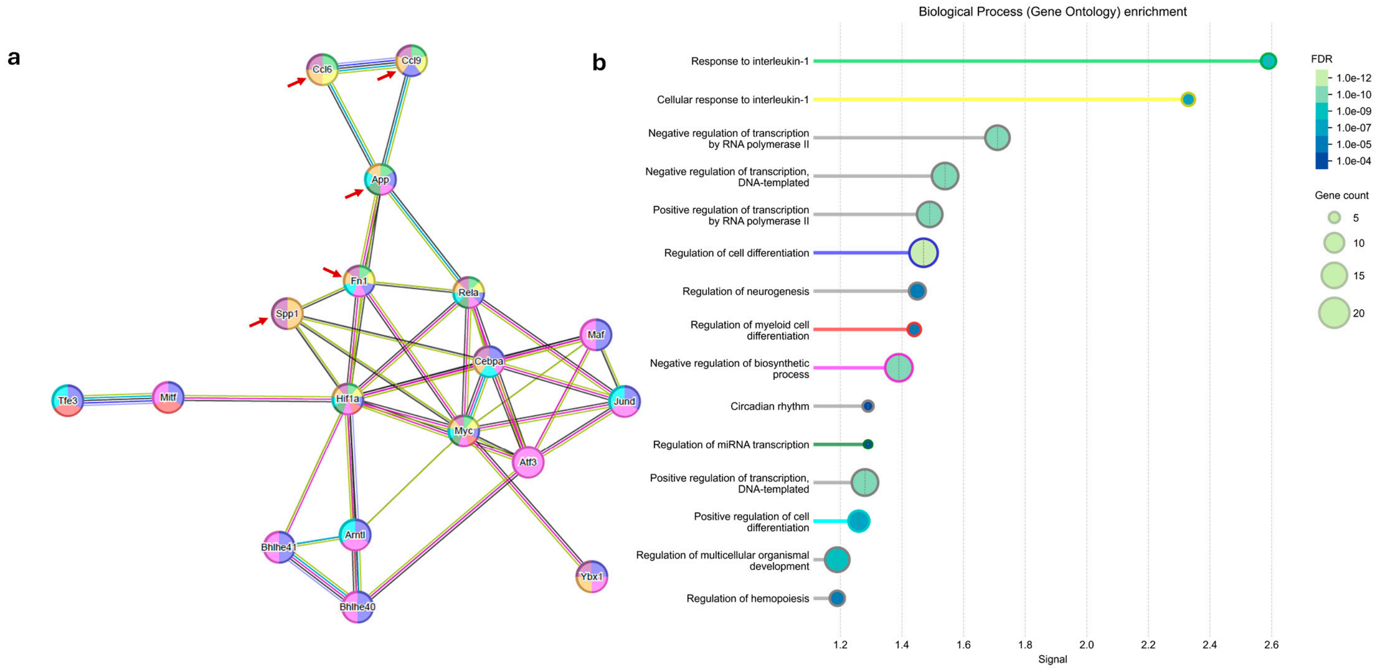

Supplementary Figure S3: Renal Macrophage Transcription Factor Biological Function Prediction: (a) Protein-protein interaction (PPI) network of the 18 upregulated TFs in Figure3c, constructed using the STRING database under medium confidence settings (minimum interaction score: 0.4) and 5 ligands we recognized in Figure3d to 3f from CellChat (including Ccl6, Ccl9, App, Spp1, Fn1: Red arrow). Nodes represent TFs(disconnected nodes in the network are hidden), and edges among nodes represent predicted associations, colored according to the type of supporting evidence: pink (experimentally determined), cyan (curated databases), green (gene neighborhood), blue (gene co-occurrence), black (co-expression), yellow (text mining), and light purple (protein homology). Nodes are colored based on their primary enriched biological process, corresponding to the highlight colors in panel b. (b) Gene Ontology (GO) Biological Process enrichment analysis for the 18 upregulated TFs and 5 ligands. Terms are sorted by "Signal“, a metric representing the weighted harmonic mean of the observed/expected ratio and the statistical significance [-log(FDR)]. Highlight colors correspond to the node colors in panel a.
